# Supplementary material for: Randomized Longitudinal Study Comparing Three Nasal Respiratory Support Modes to Prevent Intermittent Hypoxia in Very Preterm Infants
Source: Children (Basel). 2020 Oct 5;7(10):168. doi: 10.3390/children7100168 (PMC7650757; doi:10.3390/children7100168)
Supplement: Supplementary file 1 [file children-07-00168-s001.zip › Figure S1.pdf]

ANTRAG ZUR BEURTEILUNG ETHISCHER UND RECHTLICHER FRAGEN EINES MEDIZINISCHEN FORSCHUNGSVORHABENS AM MENSCHEN

Titel

Schätzung der Effektivität von zwei nasalen Atmungshilfen zur Behandlung von Frühgeborenen mit Atmungsregulationsstörungen

Beteiligte Wissenschaftler

Studienleiterin und Ansprechpartnerin der Ethik-Kommission:
Dr. med. Renate Steinfeldt
Abteilung für Neonatologie
Klinik für Kinder- und Jugendmedizin
Calwerstr. 7, 72076 Tübingen
Telefon 07071-2984750, Fax 07071-295485
e-mail: renate.steinfeldt@med.uni-tuebingen.de

Methodische Beratung:
Univ.-Prof. Dr. med. Michael S. Urschitz, EU-M.Sc.
Schwerpunkt Pädiatrische Epidemiologie
Institut für Medizinische Biometrie, Epidemiologie und Informatik
Universitätsmedizin der Johannes Gutenberg-Universität Mainz

Klinik, in der das Forschungsvorhaben durchgeführt werden soll

Neonatologische Intensivstation Neo 3 der Abteilung für Neonatologie, Klinik für Kinder- und Jugendmedizin des Universitätsklinikums Tübingen, Calwerstr.7, 72076 Tübingen

Unterschriftenliste

…..

Dr. med. Renate Steinfeldt
Studienleiterin

Finanzierung

CPAP-Geräte (Infant Flow und Stephanie) incl. pneumatische Triggerkapseln sind auf Station vorhanden.

INHALTSVERZEICHNIS

	Seite
Deckblatt	1
Inhaltsverzeichnis	2
Liste verwendeter Abkürzungen	3
Einleitung	4
Studienziel	5
Endpunkte	5
Studiendauer	5
Studiendesign	5
Studienpopulation	6
Beschreibung der Studienpopulation	
Einschlusskriterien	
Ausschlusskriterien	
Rekrutierung	
Fallzahl	
Studienablauf und Untersuchungsmethoden	7
Ablauf	
Bisherige Routineversorgung	
Studienbedingte Maßnahmen	
Erfassung von SpO_2 und Pulsfrequenz	
Weitere Datenerhebungen	
Statistische Auswertung	8
Zwischenauswertung	
Endauswertung	
Ethische Belange	9
Begründung, warum die Studie bei Minderjährigen durchgeführt werden muss	
Risiken und Nebenwirkungen	
Vorteile	
Studienabbruch	
Datenschutz	10
Literaturverzeichnis	10

Anhang: Elterninformation
Einverständniserklärung
Hinweis zum Datenschutz

LISTE VERWENDETER ABKÜRZUNGEN

AOP	apnea of prematurity
CPAP	continuous positive airway pressure
FiO_2	fraction of inspired oxygen
Hz	Hertz
(S)IMV	(synchronized) intermittent mandatory ventilation
PEEP	positive endexpiratorypressure
SpO_2	pulsoxymetrisch gemessene Sauerstoffsättigung
$tcPCO_2$	transkutan gemessener Kohlendioxidpartialdruck

Einleitung

Atmungsregulationsstörungen bei Frühgeborenen (Apnoen des Frühgeborenen, international: apnea of prematurity [AOP]) stellen ein erhebliches Problem bei unreifen Frühgeborenen dar [1]. Hierbei zeigen ca. 70% aller Frühgeborenen mit einem Gestationsalter von <34 Wochen klinisch relevante Apnoen und Hypoxien [2]. Diese Symptomatik ist eines der größten Probleme bei der Frühgeborenenbehandlung. Therapeutische Ansätze umfassen einerseits die Therapie mit Atmungsstimulantien [3], andererseits eine Atmungsunterstützung mittels nasaler Atmungshilfen. Die bisher etablierte Therapie mit Methylxanthinen (Coffein und Theophyllin) [3] ist häufig nicht ausreichend wirksam, so dass zusätzliche Maßnahmen wie die Applikation kontinuierlichen Drucks (continuous positive airway pressure, CPAP) in Form einer nasalen CPAP-Atmungshilfe oder eine (Re-)Intubation zur Anwendung kommen müssen.

Die nasale CPAP-Behandlung ist im klinischen Alltag eine etablierte Methode zur Behandlung der AOP [4]. Der theoretische Hintergrund für diese Therapie liegt in der Annahme, dass durch Blähung der Lunge, Verbesserung der funktionellen Residualkapazität und Verminderung des Atemwegswiderstandes [5] die Atmungsarbeit reduziert und durch Beeinflussung des Hering-Breuer-Reflexes [6] sowie inhibitorischer interkostaler Reflexbahnen [7] und eine Optimierung der Oxygenierung die Apnoehäufigkeit konsekutiv gesenkt wird. Es gibt jedoch nur wenige kontrollierte Studien zur Wirksamkeit dieser Therapie. In diesen wurde die Häufigkeit zentraler Apnoen offenbar nicht beeinflusst [8]; ein Effekt auf die allein für das spätere Outcome wichtigen [9] rezidivierende Hypoxien wurde nicht untersucht.

Zur CPAP-Behandlung werden meist Beatmungsgeräte, wie sie auch zur intermittierenden Überdruckbeatmung benutzt werden, eingesetzt. Ein Verfahren zur CPAP-Anwendung stellt das sog. Infant Flow System dar, bei dem CPAP über ein sog. Venturi-Prinzip generiert wird. Hierdurch soll über eine stärkere Reduktion der Atmungsarbeit im Vergleich zu konventionellen CPAP-Systemen die Häufigkeit von Atmungsregulationsstörungen beeinflusst werden [10]. Eine Neuentwicklung ist die Kombination von CPAP und nasaler Beatmung. Hierbei kommt vor allem die nasale intermittierende mandatorische Beatmung (nasal intermittent mandatory ventilation, N-IMV) zur Anwendung.

Die Vor- und Nachteile der hier aufgeführten Atmungshilfen zur AOP-Therapie und ihre Wirksamkeit wurden bislang nicht ausreichend untersucht, und es bleibt unklar, welches das effektivste Verfahren ist. In einer Vorstudie [11] konnten wir einen positiven Effekt auf intermittierende Hypoxien bei Anwendung des reinen Infant Flow-CPAP Systems im Vergleich zu 3 anderen nasalen Atmungsunterstützungs-Verfahren feststellen. Dies war insofern unerwartet, als eine nasale Beatmung (N-IMV) an sich effektiver sein sollte als die reine CPAP-Applikation. In unserer Vorstudie war N-IMV allerdings ohne Synchronisation mit den kindlichen Atmungsexkursionen angewandt worden (diese stand uns damals noch nicht zur Verfügung), was eine Erklärung für dessen vergleichsweise schlechte Wirksamkeit sein könnte. Zwischenzeitlich wird aber auch die Möglichkeit zur Synchronisation der Atemunterstützung mit der kindlichen Eigenatmung kommerziell angeboten und in unserer Abteilung eingeführt.

STUDIENZIEL

Die Antragsteller wollen die Wirksamkeit eines neuen nasalen Atmungsunterstützungs-Verfahrens auf die Häufigkeit von Hypoxien bei Frühgeborenen im Sinne einer Phase-2-Studie untersuchen. Hierbei soll ein Vergleich zwischen dem effektivsten System der letzten Studie (Infant Flow-CPAP) und einem anderen seit einigen Jahren in der Routineanwendung etablierten nasalen Atmungsunterstützungs-Regime angestellt werden. Die Bedeutung dieser Untersuchung liegt in der sich daraus ableitenden Hypothesenbildung und Fallzahlberechnung für eine zukünftige randomisiert-kontrollierte Phase-3-Studie. Untersucht werden soll das sogenannte N-SIMV-CPAP, eine nasale CPAP-Behandlung am konventionellen Respirator mit Applikation der Atmungsunterstützung über Stephan-Prongs und zusätzlicher Hintergrund-Atmungsunterstützung. Eine Synchronisierung mit der Atmung des Kindes erfolgt über eine abdominal angebrachte Druckkapsel (sog. Graseby-Kapsel); N-SIMV: f=20/min., P insp.: 15 cm H_2O, PEEP: 5 cm H_2O.

ENDPUNKTE

Primärer Zielparameter der Studie ist die Rate von Hypoxien, ausgedrückt als Ereignisse pro Stunde. Dabei werden Hypoxien definiert als Abfall der pulsoximetrischen O2-Sättigung (SpO2) auf <80%. Wir erwarten eine Abnahme der Hypoxämiehäufigkeit mit dem N-SIMV-CPAP im Vergleich zum Infant Flow-CPAP. Sekundäre Zielparameter sind die mittlere FiO2 und tcPCO2 sowie die Rate an Bradykardien (HF <80/Min.) am Ende der jeweiligen 8-stündigen Periode mit N-SIMV -CPAP im Vergleich zum Infant Flow-CPAP.

STUDIENDAUER

Die Studie ist vorerst für den Zeitraum von 2 Jahren geplant. Sollten vor Ablauf der 2 Jahre alle Probanden untersucht sein, wird die Studie vorzeitig beendet. Die Zeitdauer der Studienteilnahme pro Patient beträgt insgesamt ca. 8 Stunden.

STUDIENDESIGN

Es sollen 2 Fallserien mit je 2 Studienphasen im Vorher-Nachher Design durchgeführt werden. Es gibt insgesamt 2 Behandlungsvarianten: die Kontrollbehandlung InfantFlow-CPAP und die Testbehandlung N-SIMV. Jede der beiden Fallserien umfasst 2 Behandlungsvarianten. Fallserie 1 hat die Abfolge konventioneller CPAP=> N-SIMV, Fallserie 2 die Abfolge konventioneller CPAP => Infant Flow-CPAP. Die Zuteilung zu einer dieser beiden Studien erfolgt durch eine bereits vor Studienbeginn erstellte Randomisierungsliste, die auf computergenerierten Zufallszahlen beruht. Die Liste wird durch einen an der klinischen Versorgung nicht beteiligten Methodiker erstellt. Sie ist dem Studienleiter und den rekrutierenden Ärzten nicht bekannt. Die Randomisierung selbst erfolgt nach Rekrutierung durch Öffnung von vorgefertigten, durchnummerierten, nicht-transparenten und verklebten Briefumschlägen.

Begründungen für das gewählte Studiendesign:

1) Auf Grund der bisherigen Datenlage ist es unklar, ob die beiden Beatmungsvarianten N-SIMV-CPAP und Infant Flow als gleichwertig zu betrachten sind. Wir halten daher die Durchführung einer randomisiert-kontrollierten Studie zurzeit für nicht angemessen. Stattdessen soll zunächst ein ggf. gleichfalls vorhandener Effekt des neuen

Atmungsunterstützungsverfahrens (N-SIMV) auf die Hypoxiehäufigkeit geschätzt werden. Basierend auf diesen Daten soll dann die erfolgversprechendste Variante ausgewählt, eine Fallzahlberechnung durchgeführt und die Effektivität der Intervention in einer randomisiert-kontrollierten Studie überprüft werden.

2) In der Routineversorgung ist es üblich, Frühgeborene mit AOP zunächst mit dem konventionellen CPAP über sog. Stephan-Prongs zu behandeln. Haben die Patienten hierunter weiterhin zu viele Hypoxie-Ereignisse, wird der CPAP-Modus intensiviert. Es erfolgt die N-SIMV-Unterstützung oder der Wechsel auf den InfantFlow-CPAP mit der Hoffnung auf effektive Behandlung der AOP. Beide bislang nicht vergleichend auf ihre Effektivität untersuchten Modi werden auf der Station routinemäßig eingesetzt. Die Auswahl erfolgt nach subjektiver Präferenz des Teams und ist nicht Evidenz- oder Leitlinienbasiert. Diesen Abläufen in der Routineversorgung wird durch das hier gewählte Studiendesign Rechnung getragen. Darüber hinaus ermöglicht es eine erste Schätzung des möglichen Therapieeffekts der beiden Testinterventionen über einen Vorher-Nachher Vergleich.

STUDIENPOPULATION

Beschreibung der Studienpopulation

Die Studienpopulation umfasst frühgeborene Kinder der neonatologischen Intensivstation Neo 3 der Abteilung für Neonatologie, Klinik für Kinder- und Jugendmedizin des Universitätsklinikums Tübingen gemäß den Einschlusskriterien.

Einschlusskriterien

1) Gestationsalter bei Geburt ≤ 34 0/7 Schwangerschaftswochen
2) Gestationsalter bei Untersuchung ≤ 38 0/7 Schwangerschaftswochen
3) persistierende Apnoesymptomatik trotz Coffein- und konventioneller CPAP-Behandlung
4) Apnoescore ≥ 5 (auf Station üblicher Score zur Erfassung der Häufigkeit und Schwere von AOP, Zählung jeweils pro Schicht, d.h. über 8 Stunden)
5) Vorliegen einer schriftlichen Einwilligung des/der Erziehungsberechtigten zur Teilnahme an der Studie

Ausschlusskriterien

1) Vorliegen kongenitaler schwerer Fehlbildungen (z.B. Vitium cordis, Chromosomenanomalien)
2) Vorliegen symptomatischer Apnoen als Folge einer Sepsis, eines Krampfanfalles, einer Hypoglykämie oder einer Hirnblutung
3) Diagnose einer neuromuskulären, muskulären oder Skelett-Erkrankung

Rekrutierung

Probanden werden durch Ansprechen ihrer Eltern durch die Studienleiterin oder Oberärzte der neonatologischen Intensivstation des Universitätsklinikums Tübingen rekrutiert.

Fallzahl

Die Zahl der zu rekrutierenden Patienten wird mit der Absicht bestimmt, eine klinisch relevante Verbesserung durch zumindest eine der beiden zu testenden Interventionen im Vergleich zur Kontrollintervention nachzuweisen. Es besteht nicht die Intention, einen Unterschied zwischen den beiden Testinterventionen nachzuweisen. Auf der Basis der

Ergebnisse einer unter vergleichbaren Bedingungen bereits durchgeführten Studie [11] wird erwartet, dass sich der primäre Zielparameter beim Referenzgerät im Bereich 0 bis 36 pro Stunde Beobachtungszeit (Median 2,8; Mittelwert 6,4; Schiefe 2,7) verteilen wird. Von diesem Niveau aus soll eine individuelle Abnahme der Ereignisse um 30% aufgedeckt werden (Median 1,99; Mittelwert 4,5). Es wird erwartet, dass jeder Proband zumindest 1 Ereignis zeigen wird und dass die Messreihen untereinander mit etwa r=0,85 korrelieren (belegt durch die bereits durchgeführte Studie). Da der primäre Zielparameter nicht normal verteilt ist, wird die Fallzahl über eine durch Logarithmieren generierte Testvariable berechnet (Mittelwert 1,24; Standardabweichung 1,30; Schiefe -0,375). Eine Reduktion um 30% (Mittelwert 0,88; Standardabweichung 1,30; Schiefe -0,375) entspricht nach der Transformation und der Beachtung der Korrelation von r=0,85 einer Effektstärke von 0.5055. Dieser Effekt soll mit einer Power von mindestens 80% aufgedeckt werden. Insgesamt sollen 2 gleichwertige Hypothesentests (Alternativbehandlung 1 vs. etabliertes Verfahren und Alternativbehandlung 2 vs. etabliertes Verfahren) durchgeführt werden. Da durch die beiden Vorher-Nachher-Studien zwei unabhängige Stichproben vorliegen wird als Signifikanzniveau ein Alpha von 0,05 für jeden der beiden Tests festgelegt. Da innerhalb der beiden Vorher-Nachher-Studien aber gepaarte Daten vorliegen, wird die Fallzahlberechnung auf den t-Test für verbundene Stichproben angewendet. Aus diesen Annahmen und Vorgaben ergibt sich bei einseitigem Testen und einer Power von mindestens 80% eine Fallzahl von N=26 je Studienarm (insgesamt: 52). Die tatsächliche Power beträgt mit dieser Fallzahl 80,57%. Die Fallzahlberechnung wurde mit dem Programm G*Power Version 3.1.9.2 durchgeführt.

STUDIENABLAUF UND UNTERSUCHUNGSMETHODEN

Bisherige Routineversorgung

Patienten mit AOP erhalten routinemäßig eine nasale CPAP-Atmungsunterstützung entweder i) über das Beatmungsgerät „Stephanie“ (Fa. Stephan) oder ii) über das Infant Flow-CPAP System. Treten innerhalb eines Beobachtungsfensters von 8 Stunden (Schichtdauer) weiterhin relevante Symptome einer AOP auf, erfolgt eine Intensivierung der Therapie mit N-SIMV-CPAP oder ein Wechsel des Systems, wie unter Studiendesign beschrieben.

Studienbedingte Maßnahmen

Die oben beschriebene Routineversorgung wird in 2 Punkten geändert: i) studienbedingt soll die Zuordnung zum N-SIMV- oder Infant Flow randomisiert erfolgen; ii) die Symptome und der Schweregrad der AOP sollen über einen eigenen Studienmonitor erfasst werden.

Ablauf

Sobald sich der Bedarf für eine Therapieintensivierung abzeichnet, wird der Patient für die Studie rekrutiert. Danach wird für den zusätzlichen Studienmonitor am Fuß des Kindes ein Klebesensor angebracht, um SpO_2 und Pulsfrequenz abzuleiten. Dieser Klebesensor wird auch in der Routineversorgung zur Überwachung der Kinder eingesetzt. Der Studienmonitor wird bis zur Entscheidung für oder gegen eine Therapieintensivierung am Patienten genutzt. Erfolgt eine Entscheidung für die Therapieintensivierung, wird der Patient in eine der beiden Studienarme randomisiert. Nach Durchlauf der 8-stündigen Interventionsphase wird die Studie für den Patienten beendet und der Studienmonitor wieder entfernt. Erfolgt eine Entscheidung gegen die Therapieintensivierung, wird der Patient aus der Studie ausgeschlossen. Sollten – trotz initialem Ausschluss - die Einschlusskriterien später wieder erfüllt sein, kann der Patient ggf. wieder in die Studie eingeschlossen werden. Allerdings wird jeder Patient nur einmal randomisiert.

Erfassung von SpO_2 und Pulsfrequenz

Die Aufzeichnung der pulsoximetrischen Sauerstoffsättigung, der Pulswelle und der Pulsfrequenzerfolgt mittels des speziell für Neugeborene entwickelten Überwachungsmonitors VG 300 der Firma Getemed, Teltow, Deutschland. Für die Messung wird ein Oberflächensensor benutzt.

Weitere Datenerhebungen

Es wird eine Patientencharakteristik mit folgenden Parametern erstellt: Gestationsalter zum Geburtszeitpunkt, Gestationsalter zum Messzeitpunkt, Geschlecht, Geburtsgewicht, Gewicht bei Studienbeginn, Apnoe-Score bei Studienbeginn.

STATISTISCHE AUSWERTUNG

Zwischenauswertung

Es ist keine Zwischenauswertung vorgesehen.

Endauswertung

Vor Beginn der Endauswertung werden Patienten ausgeschlossen, bei denen mindestens eine der folgenden Bedingungen zutrifft:

1) Aufnahme in die Studie, obwohl nachträglich die Verletzung eines oder mehrerer Ein- bzw. Ausschlusskriterien festgestellt wurde.
2) Nicht durchgeführte oder unvollständig durchgeführte Therapie.
3) Keine Bestimmung des primären Zielparameters möglich.

Patienten, die aus der Auswertung ausgeschlossen werden, müssen durch einen protokollgemäß behandelten Patienten ersetzt werden. Die Auswertung beginnt, nachdem die festgelegte Anzahl an protokollgemäß behandelten Patienten eingeschlossen und der primäre Zielparameter bestimmt wurde (Auswertung nach dem „Per-Protocol" Prinzip). Für jeden Patienten wird zunächst die Anzahl der Ereignisse pro Stunde Beobachtungsdauer berechnet. Sollte sich die extreme rechtsschiefe Verteilung der primären Zielvariable in den Stichproben bestätigen, wird sie durch Logarithmieren (oder durch ein anderes adäquates Verfahren) normaltransformiert.

Die primäre Auswertung betrachtet die Effekte der Alternativbehandlungen bezogen auf die Standardbehandlung getrennt in beiden Studienarmen im Sinne zweier unabhängiger „Vorher-Nachher" Studien. Dazu werden in jedem Studienarm die Mittelwerte der transformierten Testvariable mittels t-Test für verbundene Stichproben verglichen. Da es aufgrund des Studiendesigns zwei voneinander unabhängige Stichproben gibt, wird für jeden der beiden Hypothesentests ein p-Wert von 0,05 als statistisch signifikant angesehen. Sollte sich ein signifikanter Therapieeffekt nachweisen lassen, wird der Effekt als Mittelwert der individuellen Differenzen und dem 95% Konfidenzintervall dargestellt.

Alle anderen erhobenen sekundären Zielparameter werden vergleichend statistisch beschrieben. Statistische Tests in Zusammenhang mit diesen Zielvariablen haben einen rein deskriptiven Charakter und können nicht für Signifikanzaussagen herangezogen werden.

ETHISCHE BELANGE

Begründung, warum die Studie bei Minderjährigen durchgeführt werden muss

Die Atmungsstörung, die im Rahmen dieser Studie untersucht werden soll, tritt nur bei frühgeborenen Kindern auf. Daher ist es nicht möglich, diese Studie bei Erwachsenen bzw. einwilligungsfähigen Patienten durchzuführen.

Risiken und Nebenwirkungen

Die gewählten Untersuchungsverfahren sind nicht invasiv, ungefährlich und belasten den Patienten so gut wie nicht. Bei den unterschiedlichen CPAP-Behandlungsformen handelt es sich um allgemein akzeptierte und in der Routineanwendung etablierte Standardtherapien, die nach dem derzeitigen Kenntnisstand als gleichwertig anzusehen sind. Entsprechend ist die Behandlung in den verschiedenen Untersuchungsgruppen ebenfalls als gleichwertig zu beurteilen. Sowohl die nasale CPAP-Behandlung mit den o.g. Geräten als auch die atemanaleptische Therapie mit Coffein haben auf unserer Frühgeborenenstation seit Jahren einen festen Platz in der Behandlung der Apnoesymptomatik. Alle in der Studie verwendeten Medizinprodukte sind zugelassen und werden nur von Personen betrieben, angewendet und instand gehalten, die dafür die erforderliche Ausbildung und Erfahrung besitzen.

Vorteile

Es bestehen durch die Studienteilnahme keine Vorteile für die Patienten.

Abbruchkriterien

Die Studie wird in folgenden Fällen für den Probanden abgebrochen:

1) Die Eltern oder eine sorgeberechtigte Person ziehen mündlich oder schriftlich ihr Einverständnis zurück
2) Notwendigkeit einer Veränderung der Coffeindosis oder Beginn einer Doxaprambehandlung
3) Notwendigkeit einer Intubation und maschineller Beatmung
4) Notwendigkeit einer Reanimation

Es gibt keine Kriterien zum vorzeitigen Abbruch der gesamten Studie.

DATENSCHUTZ

Probanden werden nur dann in diese Untersuchung eingeschlossen, wenn die Eltern oder (eine) sorgeberechtigte Person/en ihr informiertes Einverständnis schriftlich erklären. Da den Eltern das Ergebnis der Überwachung nicht mitgeteilt werden soll, ist die Erfassung von personenidentifizierenden Angaben der Kinder (Name, Geburtsdatum, Anschrift) nicht notwendig. Für die weitere wissenschaftliche Auswertung im Sinne der oben beschriebenen Fragestellungen werden alle Daten eines Probanden unter einer fortlaufenden und nicht sprechenden Code-Nummer zusammengeführt, elektronisch gespeichert und statistisch ausgewertet. Nach der wissenschaftlichen Auswertung

werden keine Daten oder Ergebnisse veröffentlicht, aus denen die Identität von Probanden dieser Studie hervorgehen würde.

Die im Rahmen dieser Studie gesammelten Daten werden ausschließlich zum Zwecke der beschriebenen wissenschaftlichen Fragestellungen gesammelt und ausgewertet und nicht an Dritte weitergegeben. Es wird dafür Sorge getragen, dass nur an der Studie beteiligte Personen Zugang zu den Daten und Unterlagen erhalten und die ärztliche Schweigepflicht gewahrt bleibt. Alle Unterlagen mit Angaben über Probanden werden spätestens 10 Jahre nach Abschluss der Studie vernichtet.

LITERATURVERZEICHNIS

1. Miller, M. J., Martin, R. J. (1992). Apnea of prematurity. ClinPerinatol. 19:789-808

2. Barrington, K., Finer, N. (1991). The natural history of the appearance of apnea of prematurity. Pediatr Res. 29:372-375

3. Henderson-Smart, D. J., Steer, P. (2001). Methylxanthine treatment for apnea in preterm infants. Cochrane Database Syst Rev. CD000140

4. Lemyre, B., Davis, P. G., De Paoli, A. G. (2003). Nasal intermittent positive pressure ventilation (NIPPV) versus nasal continuous positive airway pressure (NCPAP) for apnea of prematurity. Cochrane Database Syst Rev. CD002272

5 Klausner, J. F., Lee, A. Y., Hutchison, A. A. (1996). Decreased imposed work with a new nasal continuous positive airway pressure device. PediatrPulmonol. 22:188-194

7. Hagan, R., Bryan, A. C., Bryan, M. H., Gulston, G. (1977). Neonatal chest wall afferents and regulation of respiration. J Appl Physiol. 42:362-367

8. Courtney, S. E., Pyon, K. H., Saslow, J. G., Arnold, G. K., Pandit, P. B., Habib, R. H. (2001) Lung recruitment and breathing pattern during variable versus continuous flow nasal continuous positive airway pressure in premature infants: an evaluation of three devices. Pediatrics. 107:304-308

9. Poets CF, Roberts RS, Schmidt B, et al; Canadian Oxygen Trial Investigators (2015). Association between intermittent hypoxemia or bradycardia and late death or disability in extremely preterm infants. JAMA 314:595-603

10. Pandit, P. B., Courtney, S. E., Pyon, K. H., Saslow, J. G., Habib, R. H. (2001). Work of breathing during constant- and variable-flow nasal continuous positive airway pressure in preterm neonates. Pediatrics. 108:682-685

11. Pantalitschka, T., Sievers, J., Urschitz, M. S., Herberts, T., Reher, C., Poets, C. F. (2009). Randomised crossover trial of four nasal respiratory support systems for apnoea of prematurity in very low birthweight infants. Arch Dis Child Fetal Neonatal Ed. 94:F245-F248
