# Supplementary material for: Randomized Longitudinal Study Comparing Three Nasal Respiratory Support Modes to Prevent Intermittent Hypoxia in Very Preterm Infants
Source: Children (Basel). 2020 Oct 5;7(10):168. doi: 10.3390/children7100168 (PMC7650757; doi:10.3390/children7100168)
Supplement: Supplementary file 1 [file children-07-00168-s001.zip › Figure S2.docx]

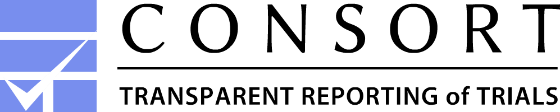


**Randomized longitudinal study comparing 3 nasal respiratory support modes to prevent intermittent hypoxia in very preterm infants**

**
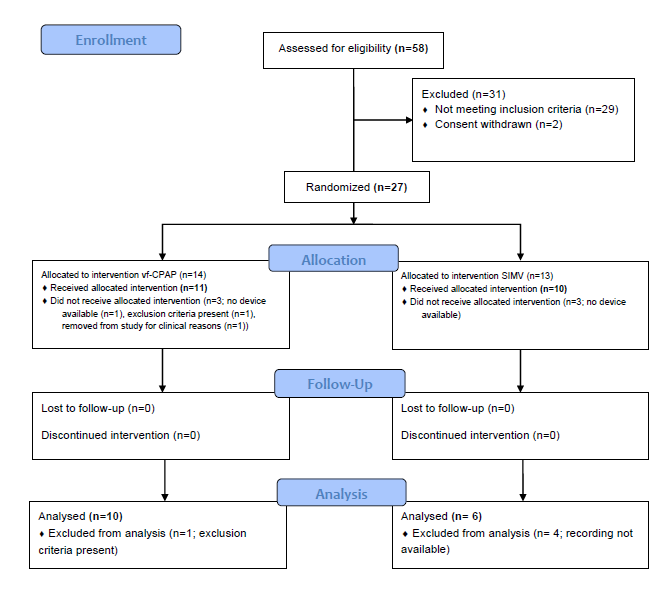
**
